# Supplementary material for: aMeta: an accurate and memory-efficient ancient metagenomic profiling workflow
Source: Genome Biol. 2023 Oct 23;24:242. doi: 10.1186/s13059-023-03083-9 (PMC10591440; doi:10.1186/s13059-023-03083-9)
Supplement: Supplementary file 1 — Additional file 1: Supplementary figures Fig. S1-24, additional figures with technical information not included in the main text. [file 13059_2023_3083_MOESM1_ESM.docx]

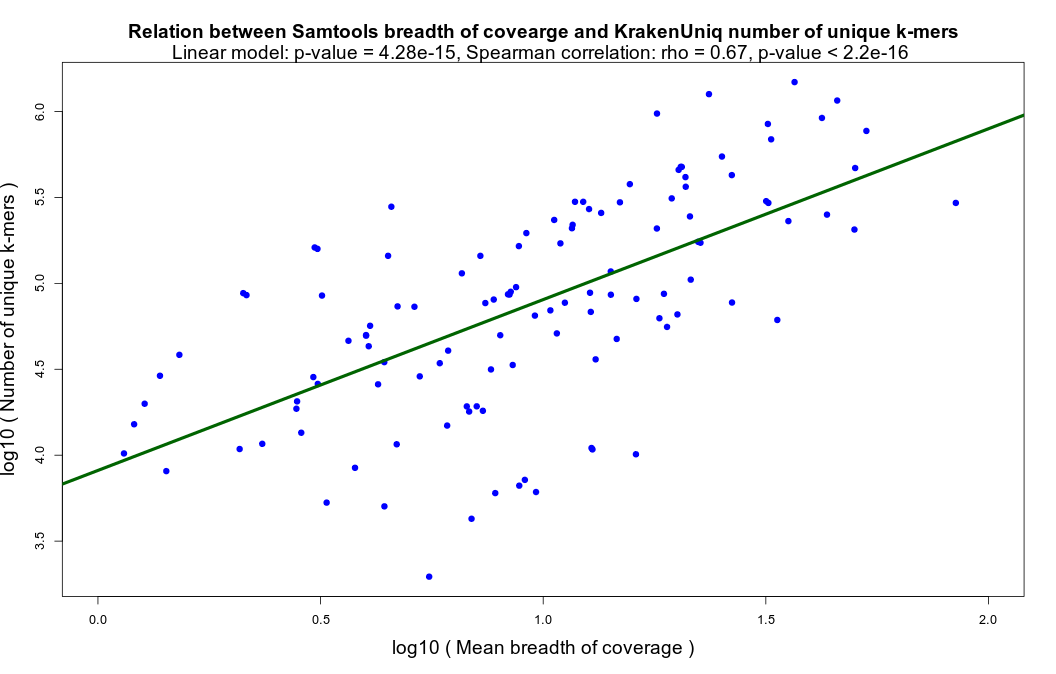


**Fig. S1.** The number of unique *k*-mers reported by KrakenUniq is strongly (significantly) correlated with the breadth of coverage computed via the *samtools depth* command from SAMtools [29] for each species detected by aMeta in the simulated ancient metagenomic samples.


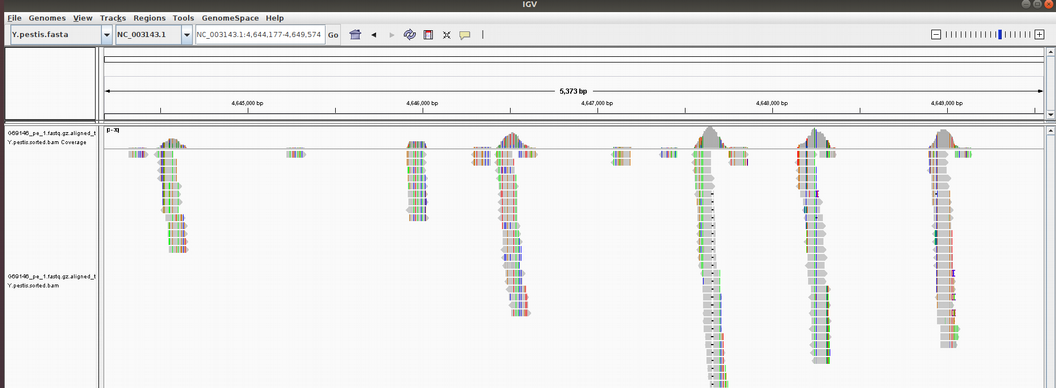


**Fig. S2.** Visualization of false-positive alignments of modern metagenomic reads to *Yersinia pestis* reference genome in IGV [30]. Metagenomic reads from a stool sample taken from a modern infant (who unlikely had a plague) were mapped against a *Y. pestis* CO92 reference genome alone, which resulted in a number of reads aligned unevenly to regions conserved across bacterial organisms.


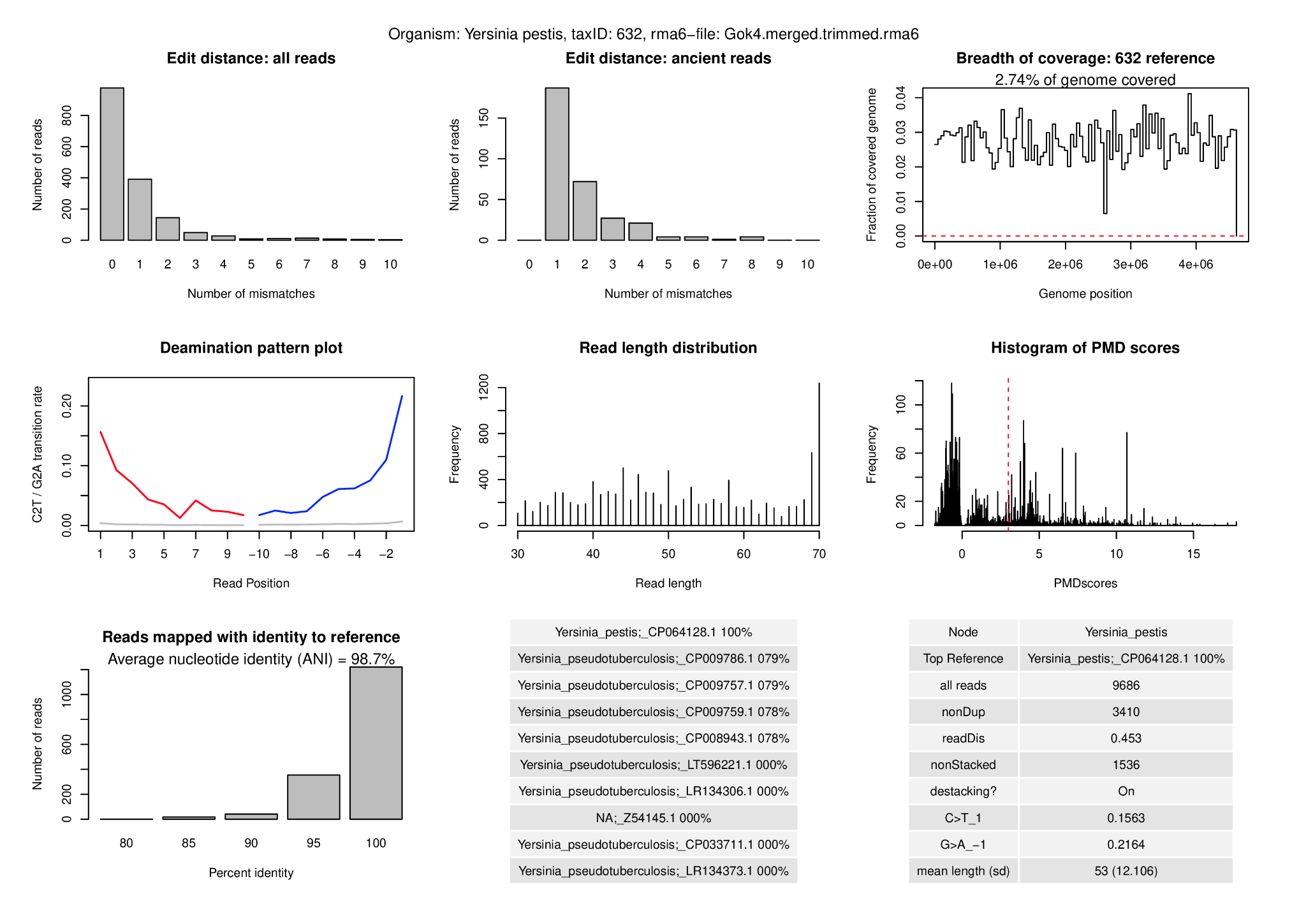


**Fig. S3.** Authentication output from aMeta for *Yersinia pestis* found in Gökhem 4 (Gok4) individual [5].


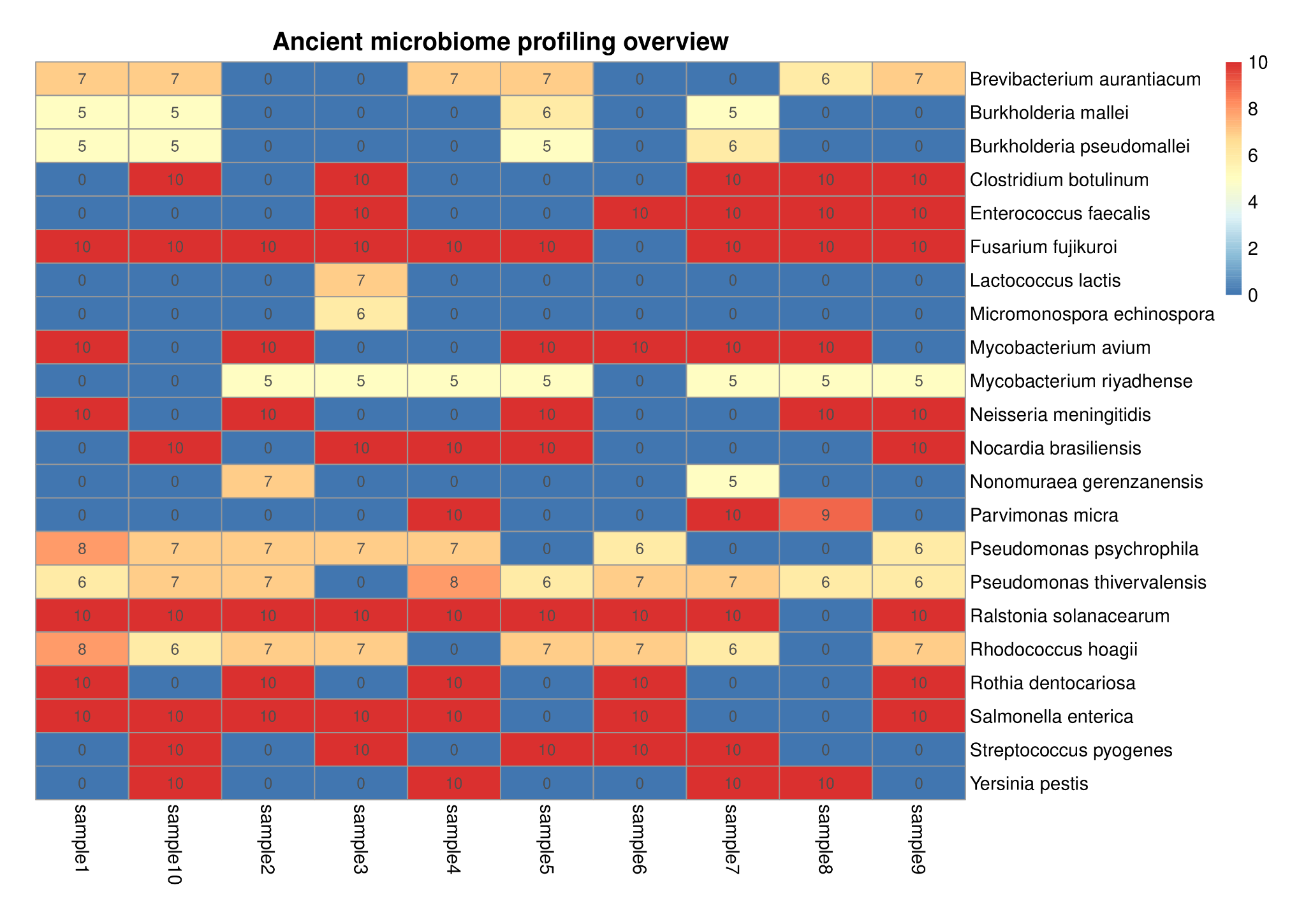


**Fig. S4.** Heatmap overview of authentication scores produced by aMeta for each detected microbe in each simulated sample.


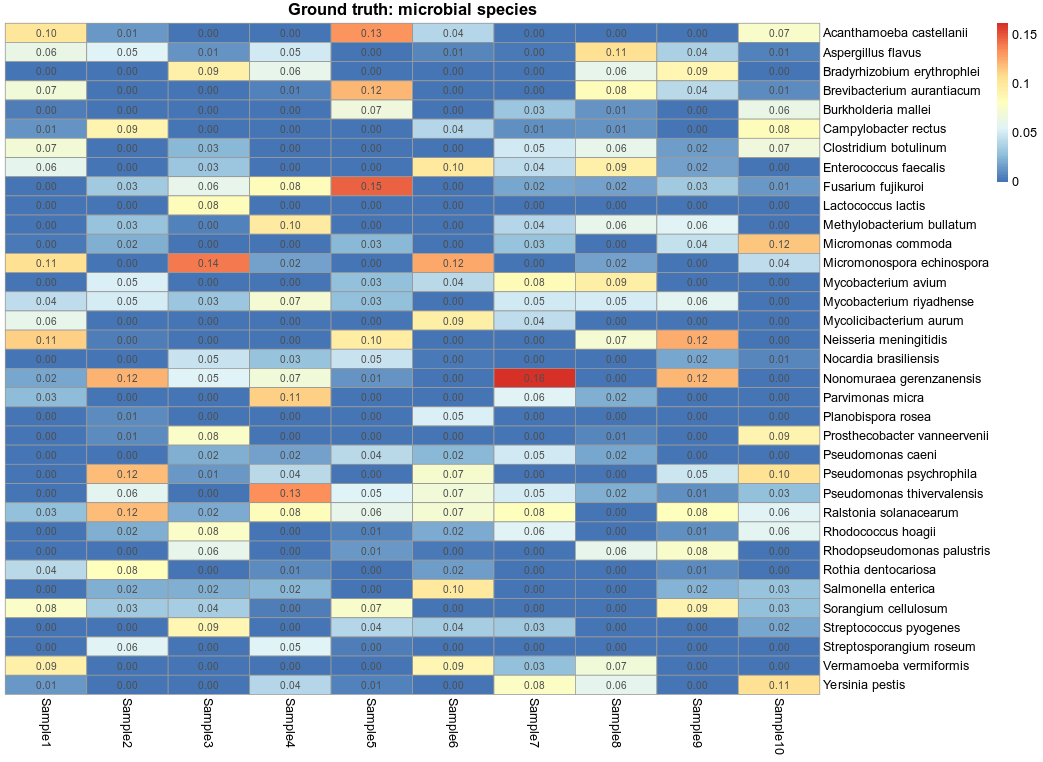


**Fig. S5.** Heatmap of ground truth microbial abundance in each simulated sample. The elements of the matrix correspond to the simulated fractions of microbes per metagenomic sample. Human reads are ignored.


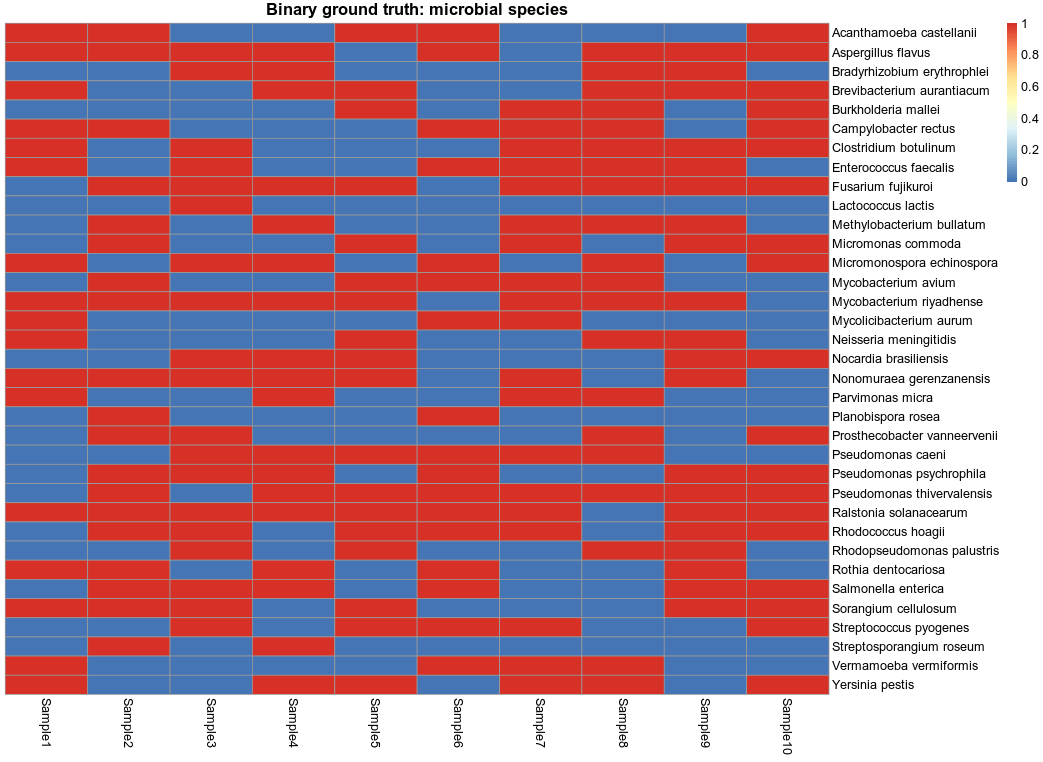


**Fig. S6.** Heatmap of binarized (0 – absent, 1 – present) ground truth microbial abundance in each simulated sample.


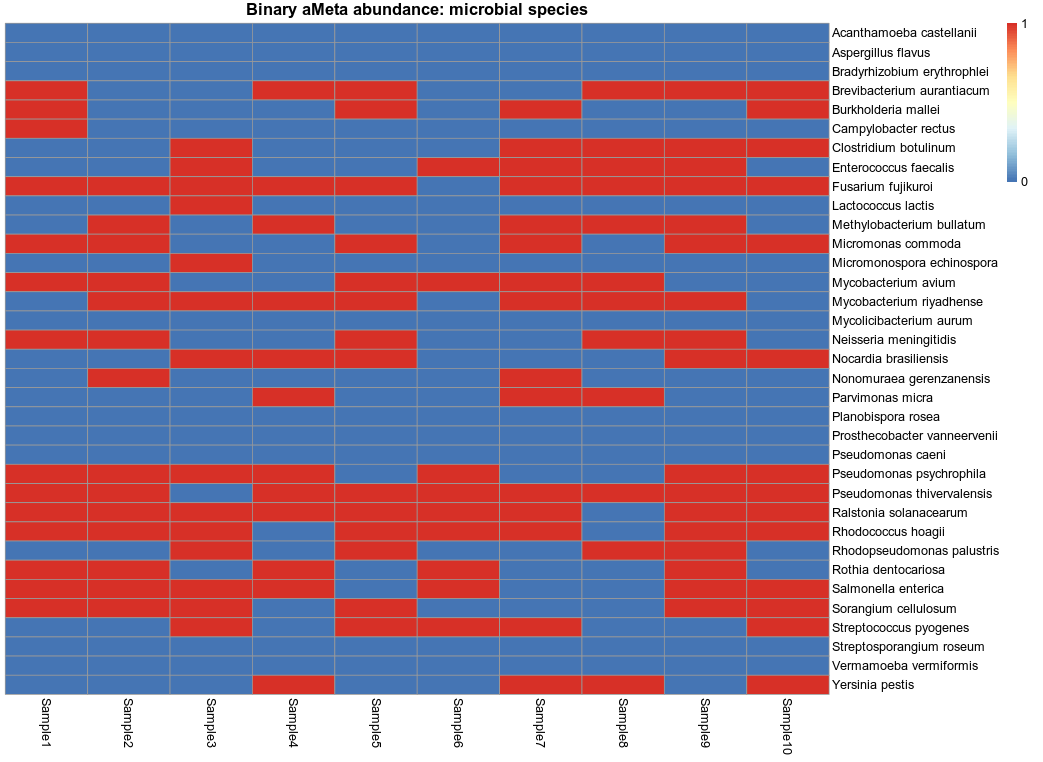


**Fig. S7.** Heatmap of binarized (0 – absent, 1 – present) microbial abundance reconstructed by aMeta in simulated samples.


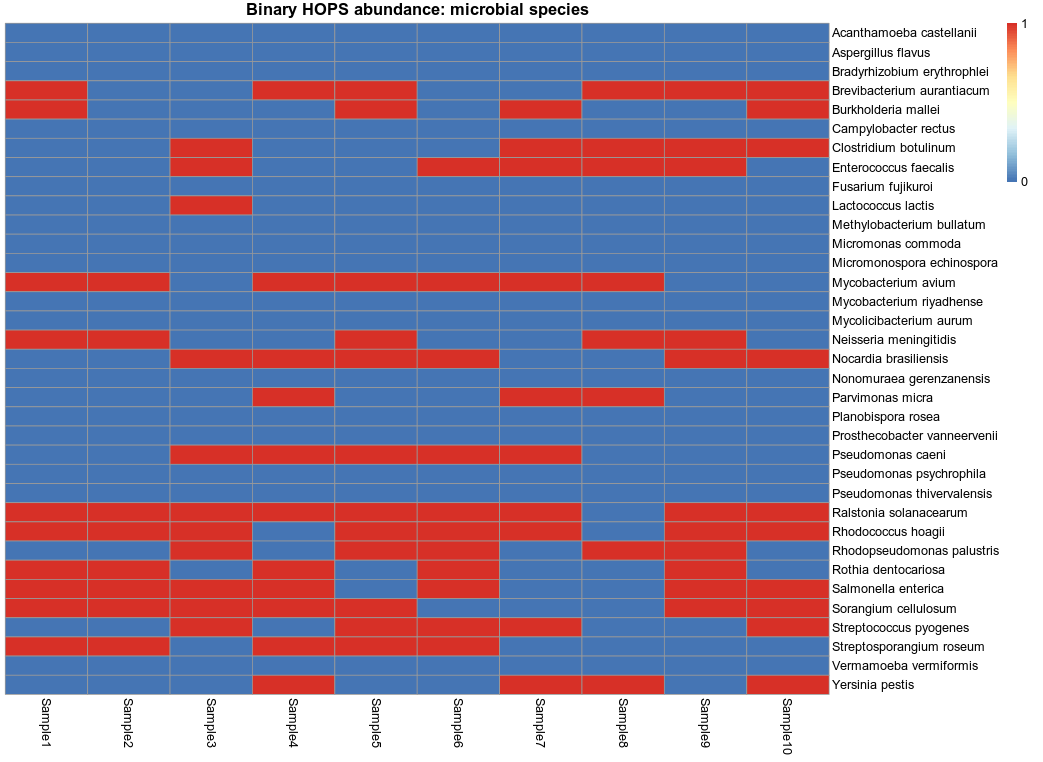


**Fig. S8.** Heatmap of binarized (0 – absent, 1 – present) microbial abundance reconstructed by HOPS in simulated samples.


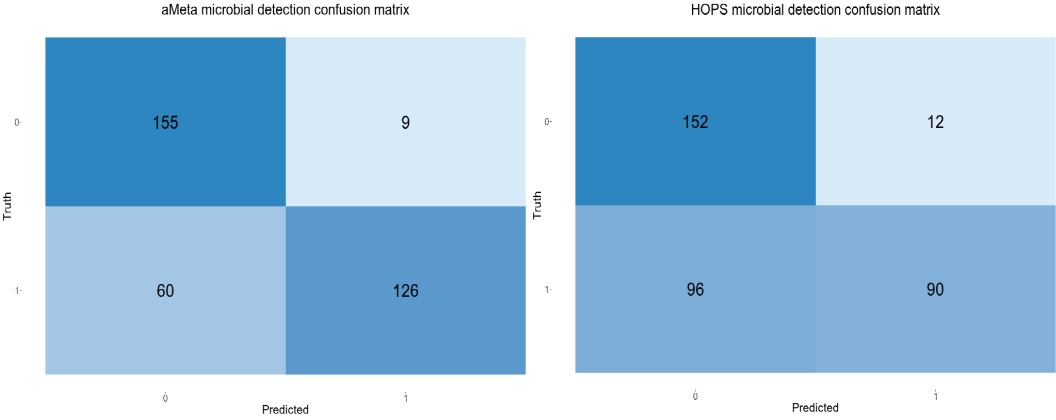


**Fig. S9.** Confusion matrix of microbial reconstruction by aMeta and HOPS: 0 – microbe absent, 1 – microbe present. The accuracy of microbial detection with aMeta is 80%, left panel, and the accuracy of microbial detection with HOPS is 69%, right panel.


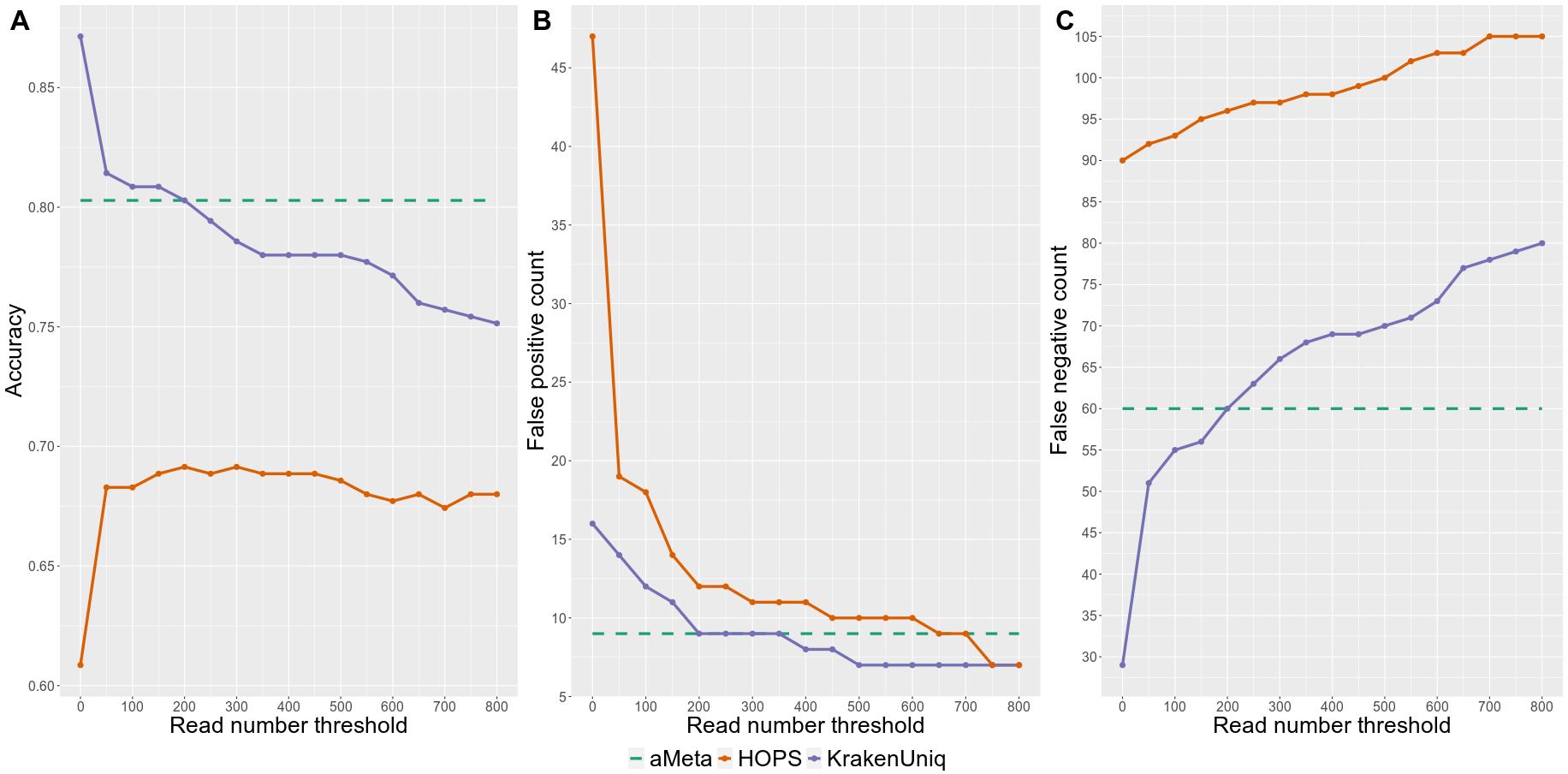


**Fig. S10.** Microbial reconstruction accuracy (A), as well as false-positive (B), and false-negative C), counts computed in simulated samples by aMeta and HOPS. aMeta demonstrates consistently higher accuracy and lower numbers of false-positive and false-negative counts compared to HOPS in a wide range of read number thresholds varying from 0 to 800 assigned reads.


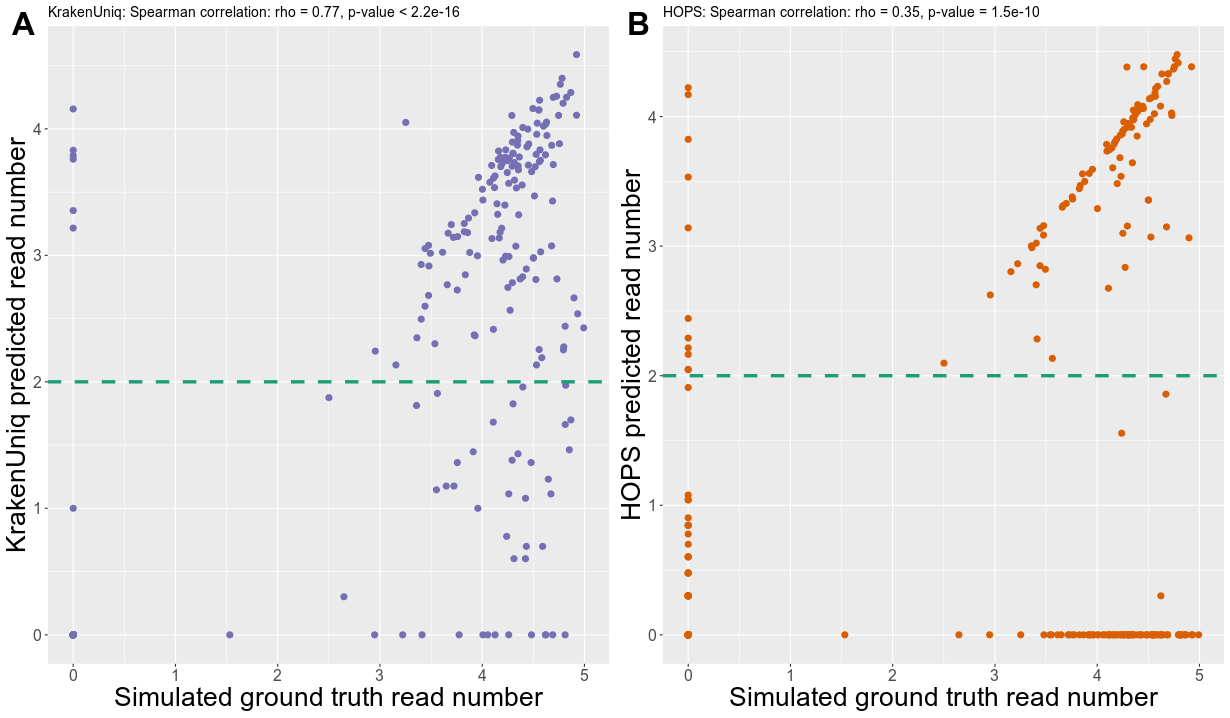


**Fig. S11.** Comparison of simulated and reconstructed read counts for aMeta / KrakenUniq (A), and HOPS (B). Horizontal dashed lines mark a reasonable read count threshold (~100-300 reads) that can be applied to KrakenUniq and HOPS abundance matrices. The axes of the plot are log10-scaled. HOPS demonstrates a higher dropout, i.e. high false-positive and false-negative counts, and poorer correlation with the ground truth.


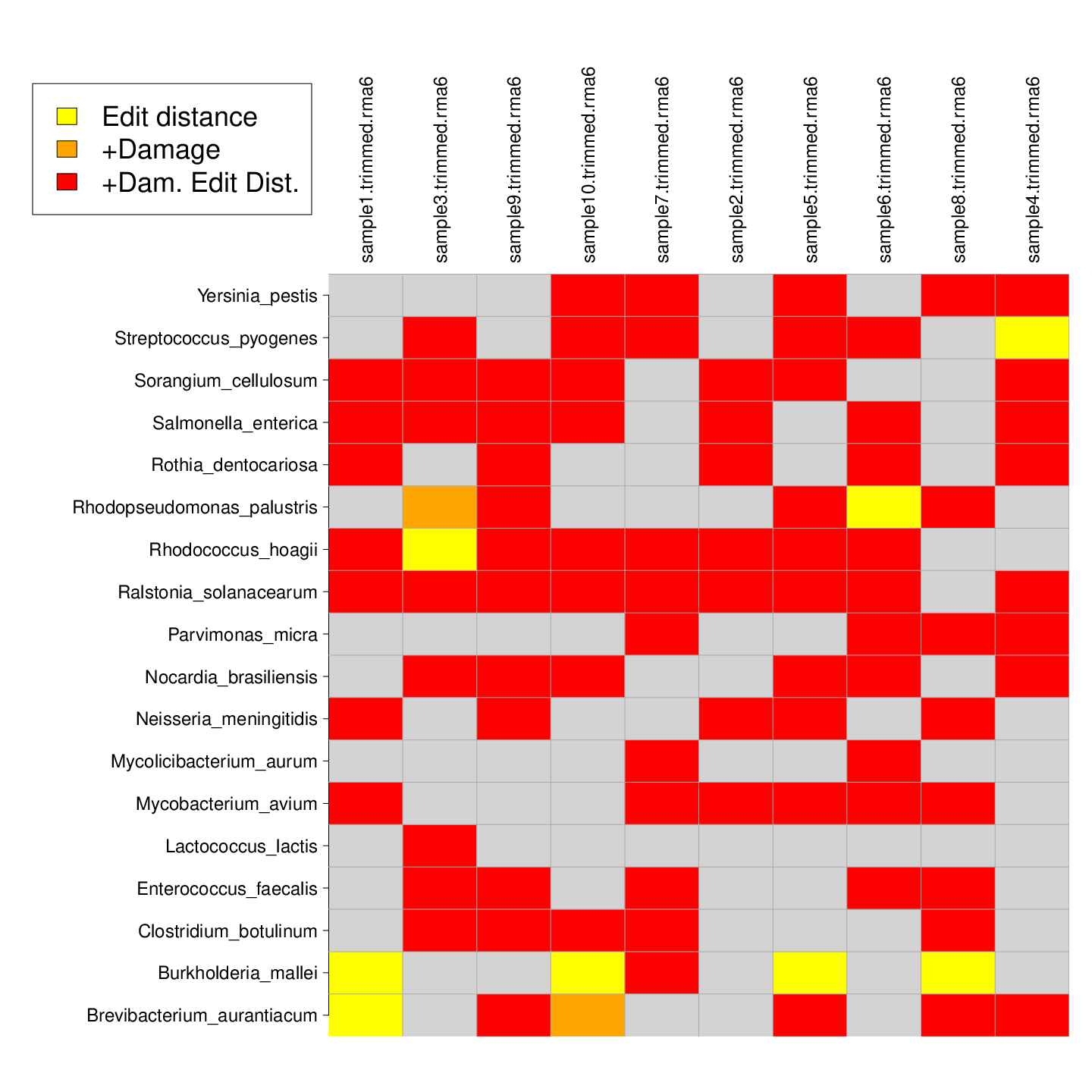


**Fig. S12.** Heatmap of HOPS authentication scores that shows different levels of confidence of ancient microbial presence in simulated data. *Rhodopseudomonas palustris, Rhodococcus hoagii, Lactococcus lactis, Brevibacterium aurantiacum, Burkholderia mallei* were simulated modern, while the other species on the y-axis of the heatmap were simulated ancient.


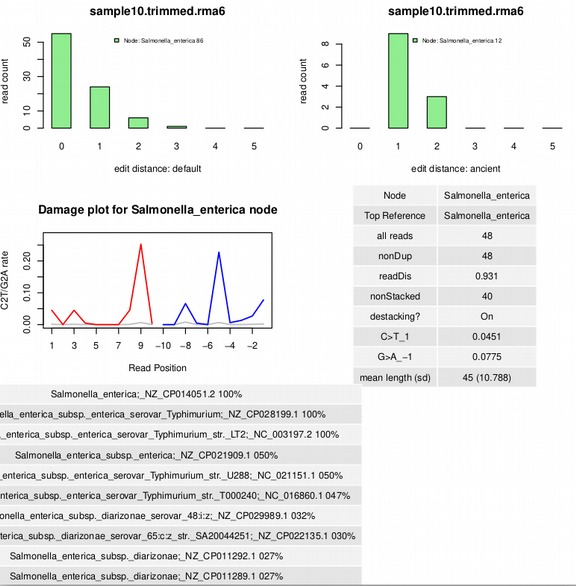


**Fig. S13.** Authentication output from HOPS for *Salmonella enterica* that was simulated to be ancient in sample 10.


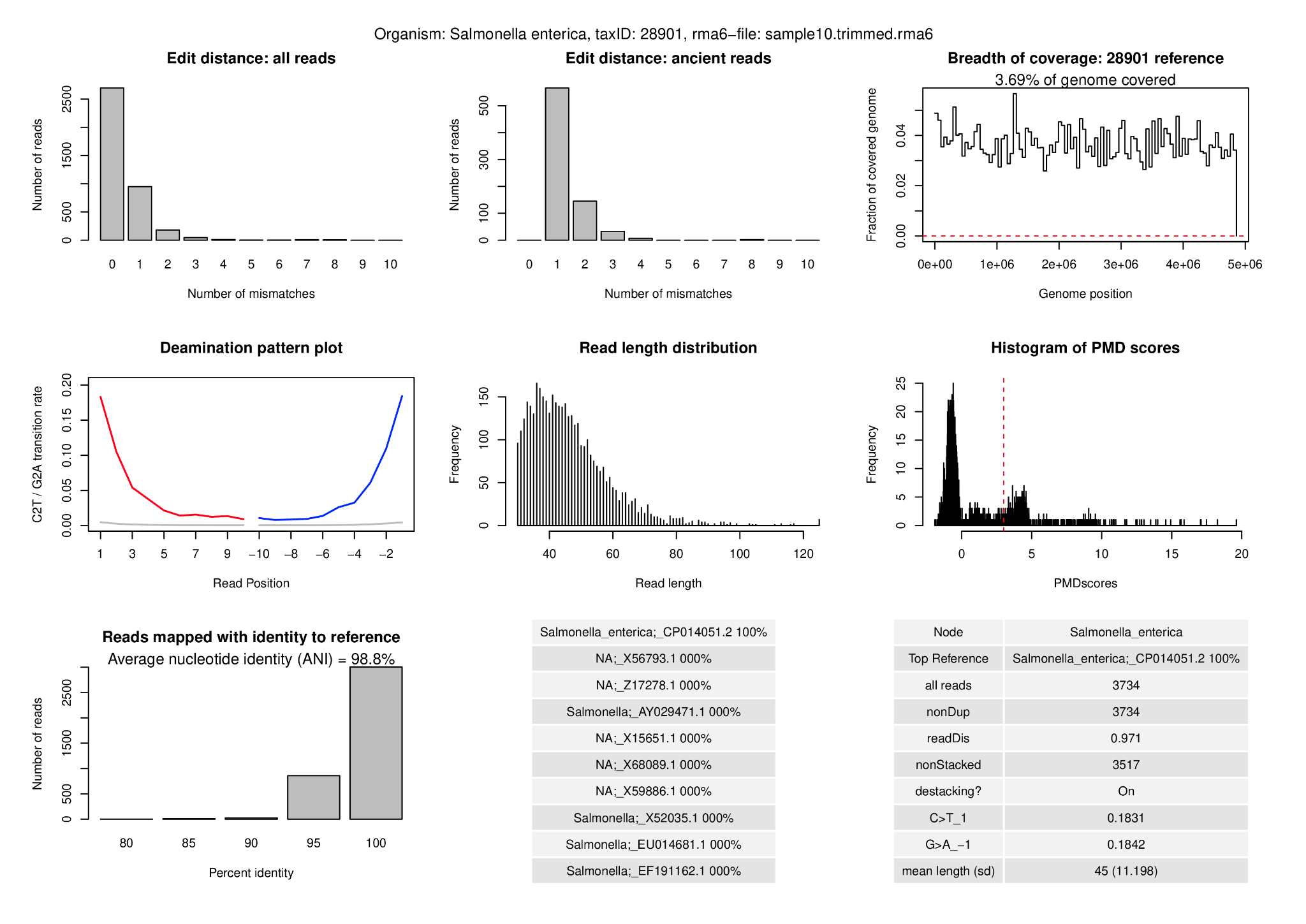


**Fig. S14.** Authentication output from aMeta for *Salmonella enterica* that was simulated to be ancient in sample 10.


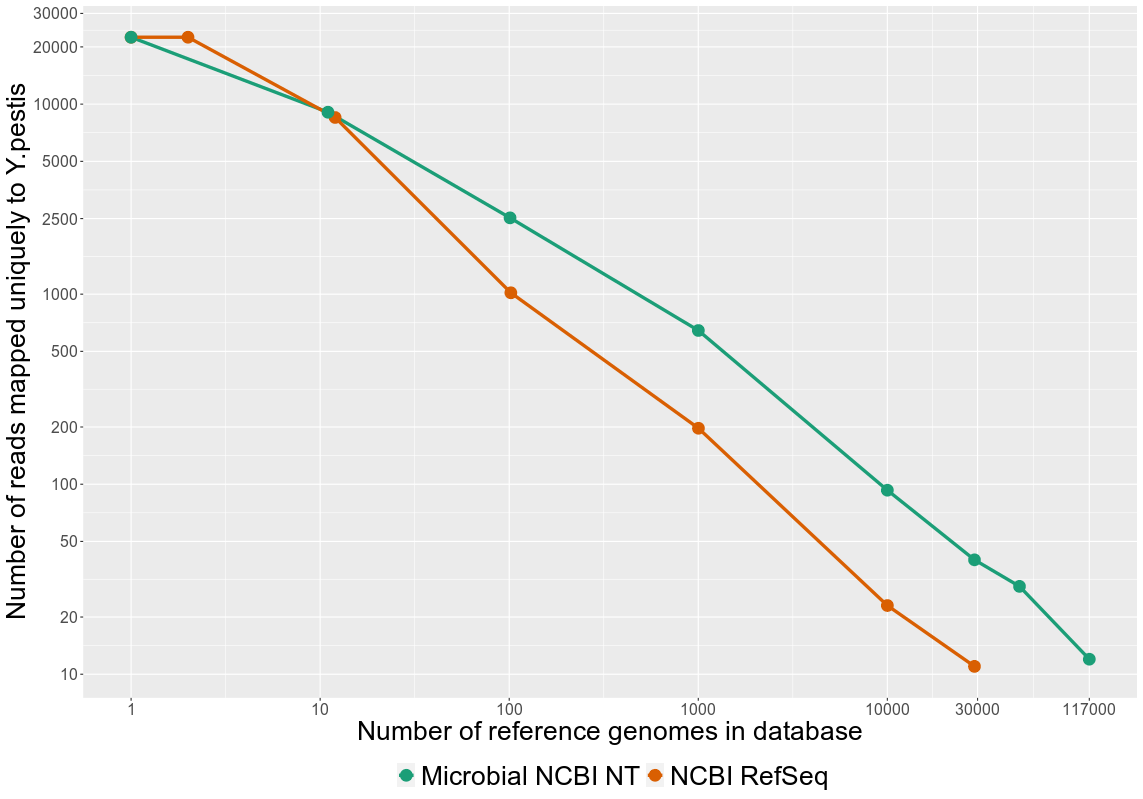


**Fig. S15.** Replication of the decreasing profile, observed in Figure 9, for the number of (misaligned) reads mapped uniquely to *Y.pestis* for the case of sampling random bacteria from the Microbial NCBI NT database.


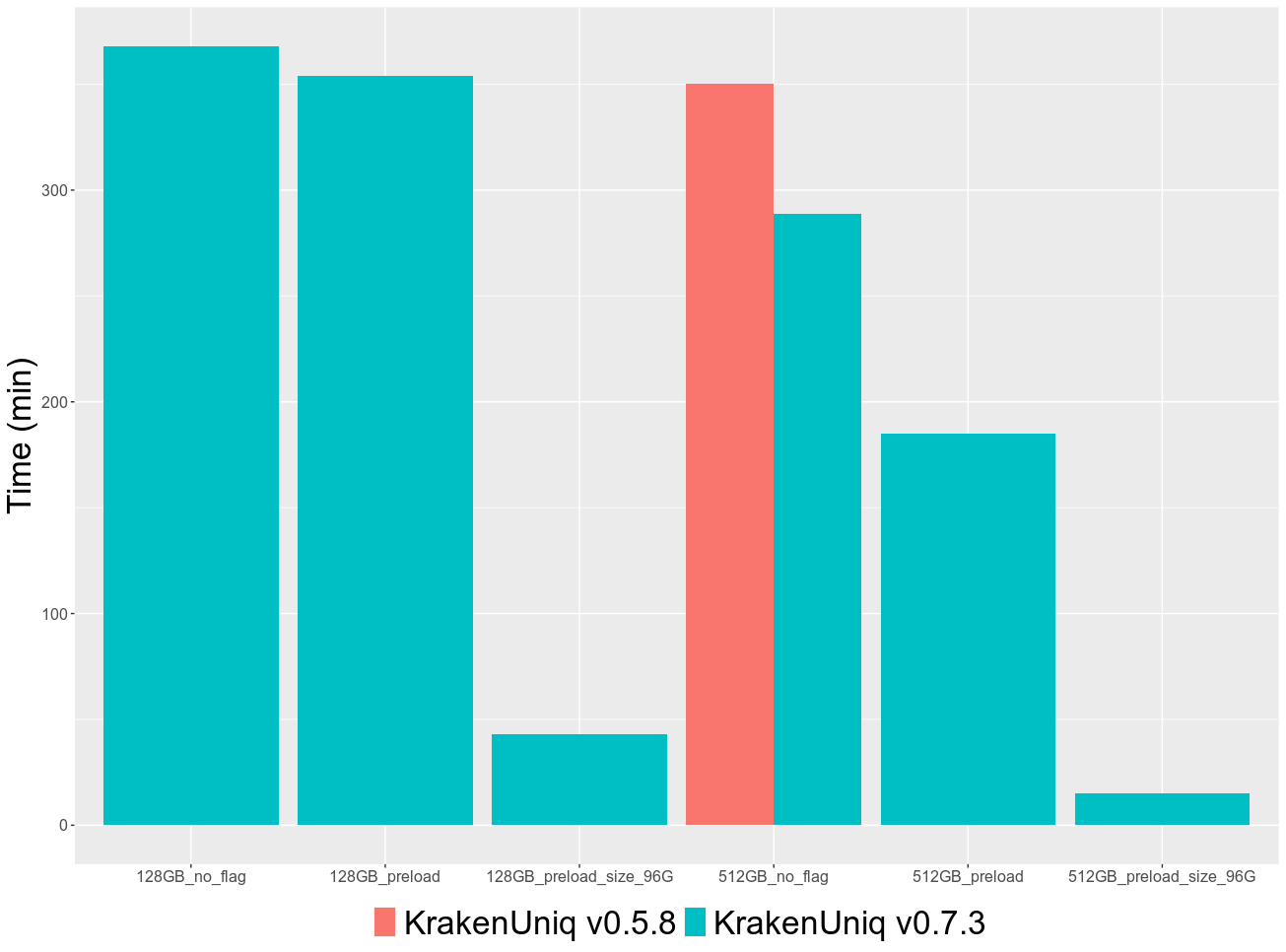


**Fig. S16.** Benchmarking KrakenUniq memory requirements for version 0.5.8 and low-memory version 0.7.3. The database used was ~450 GB in size and could only be fit into a 512 GB memory computer node when using the version 0.5.8. However, database chunking implemented in v0.7.3 allowed processing the same data on 128 GB RAM computer node, and nearly 10 times faster.


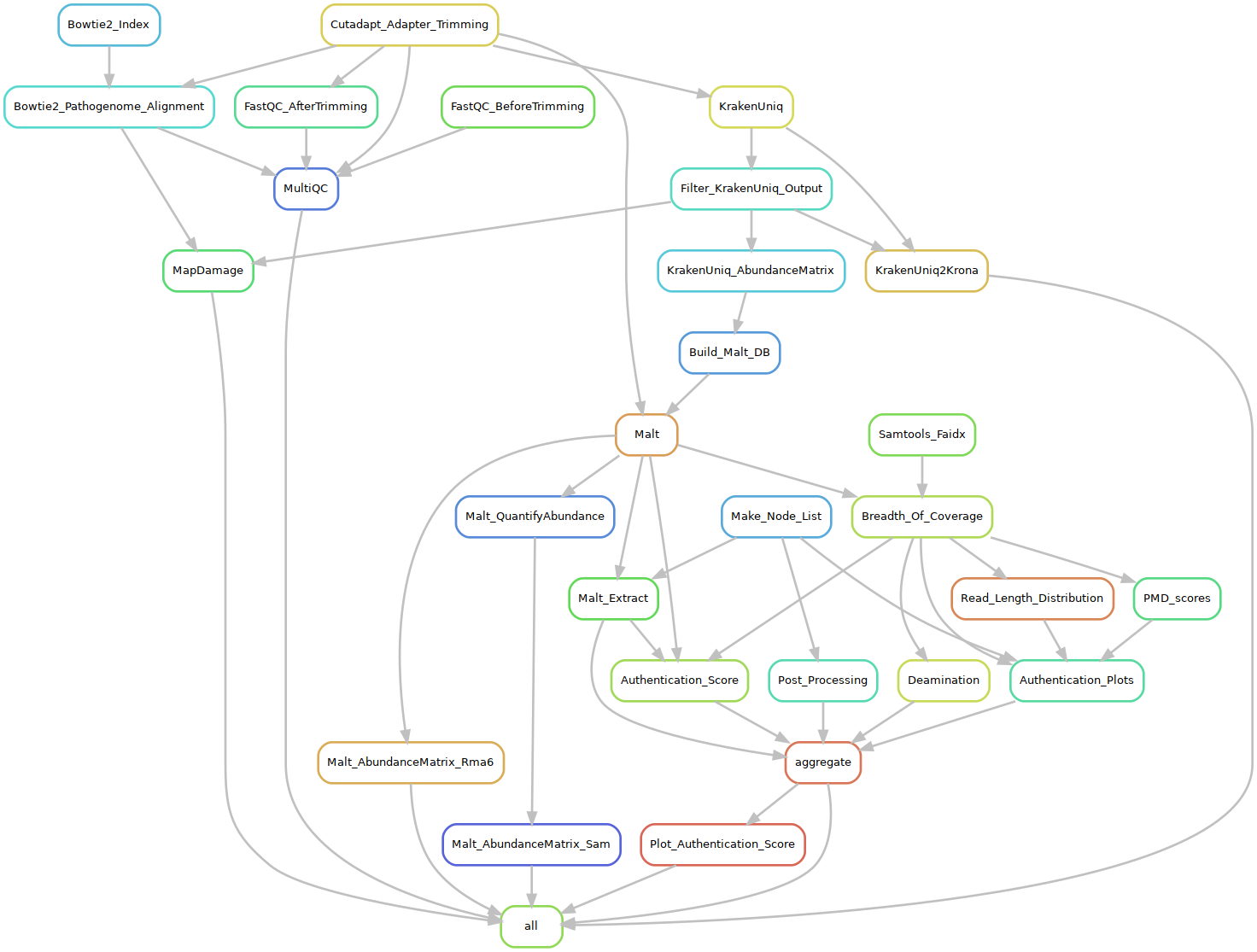


**Fig. S17.** Rule-graph (DAG) of a typical project run via the aMeta workflow.


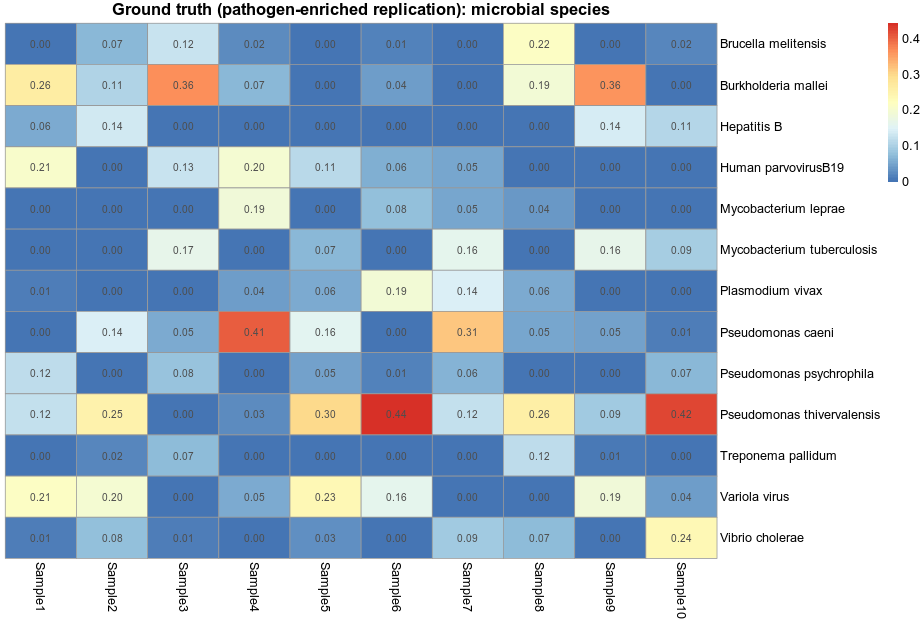


**Fig. S18.** Heatmap demonstrating ground truth microbial abundance in simulated pathogen-enriched dataset.


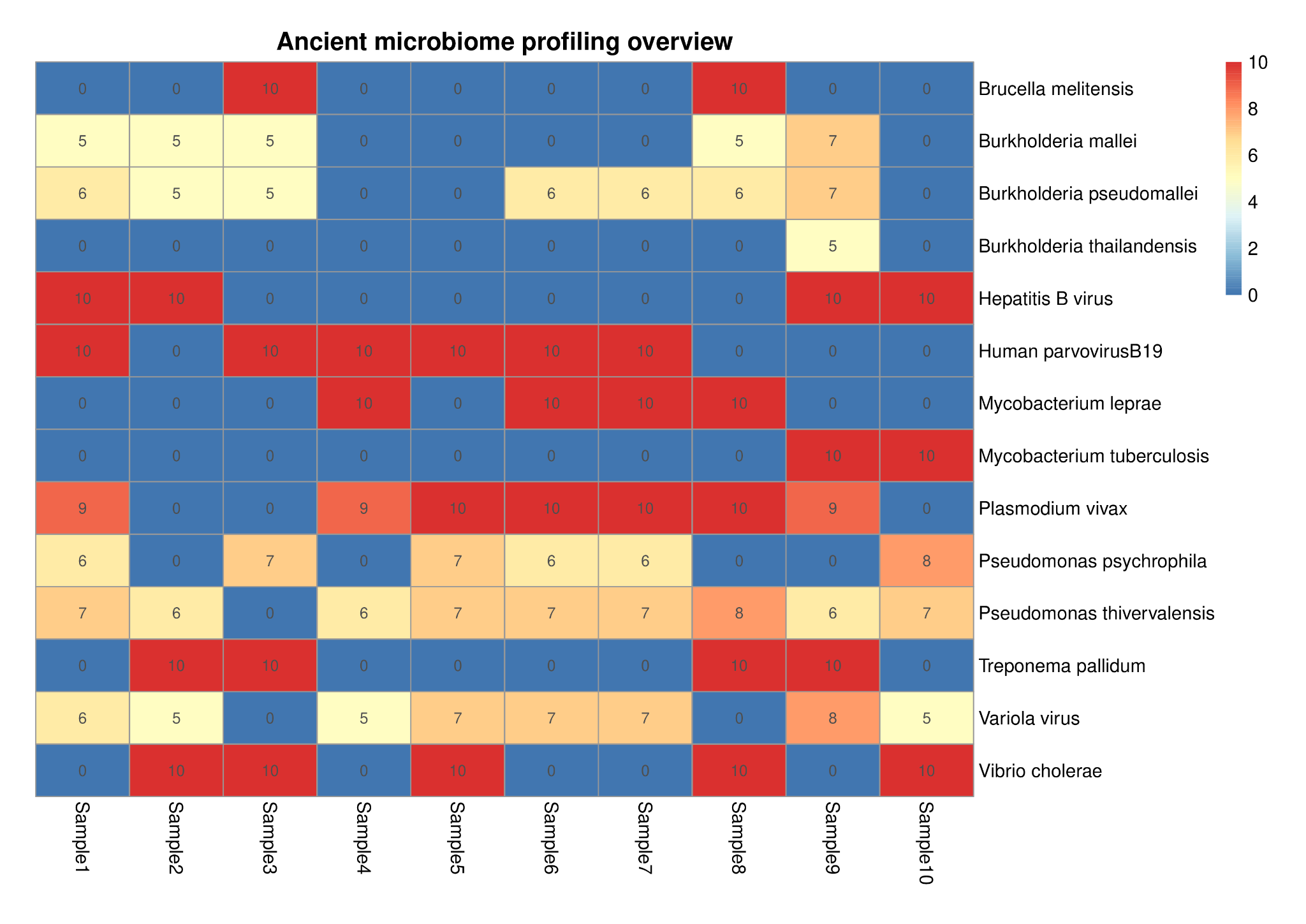


**Fig. S19.** Authentication heatmap overview of aMeta for simulated pathogen-enriched ancient metagenomic dataset.


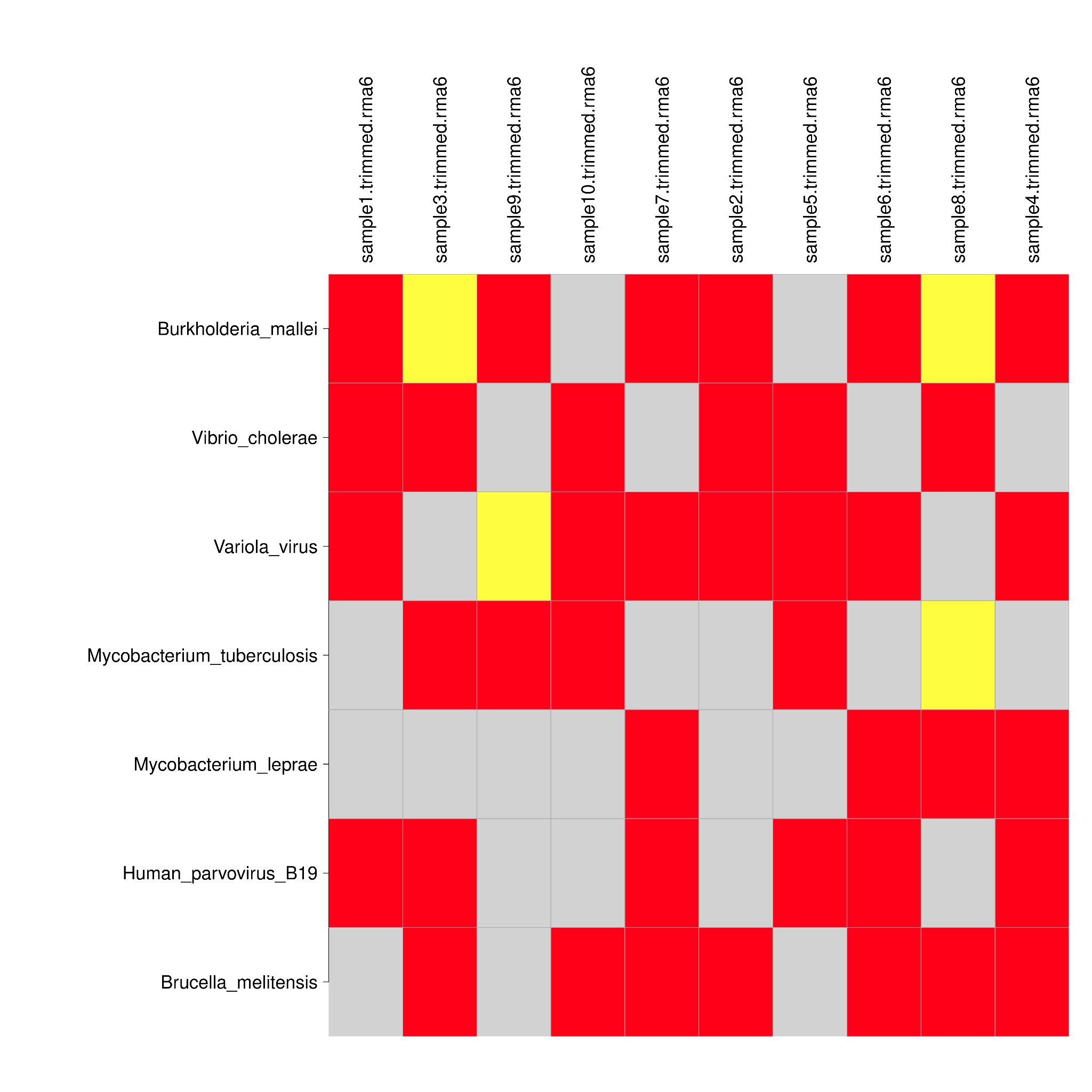


**Fig. S20.** Authentication heatmap overview of HOPS for the simulated pathogen-enriched ancient metagenomic dataset.


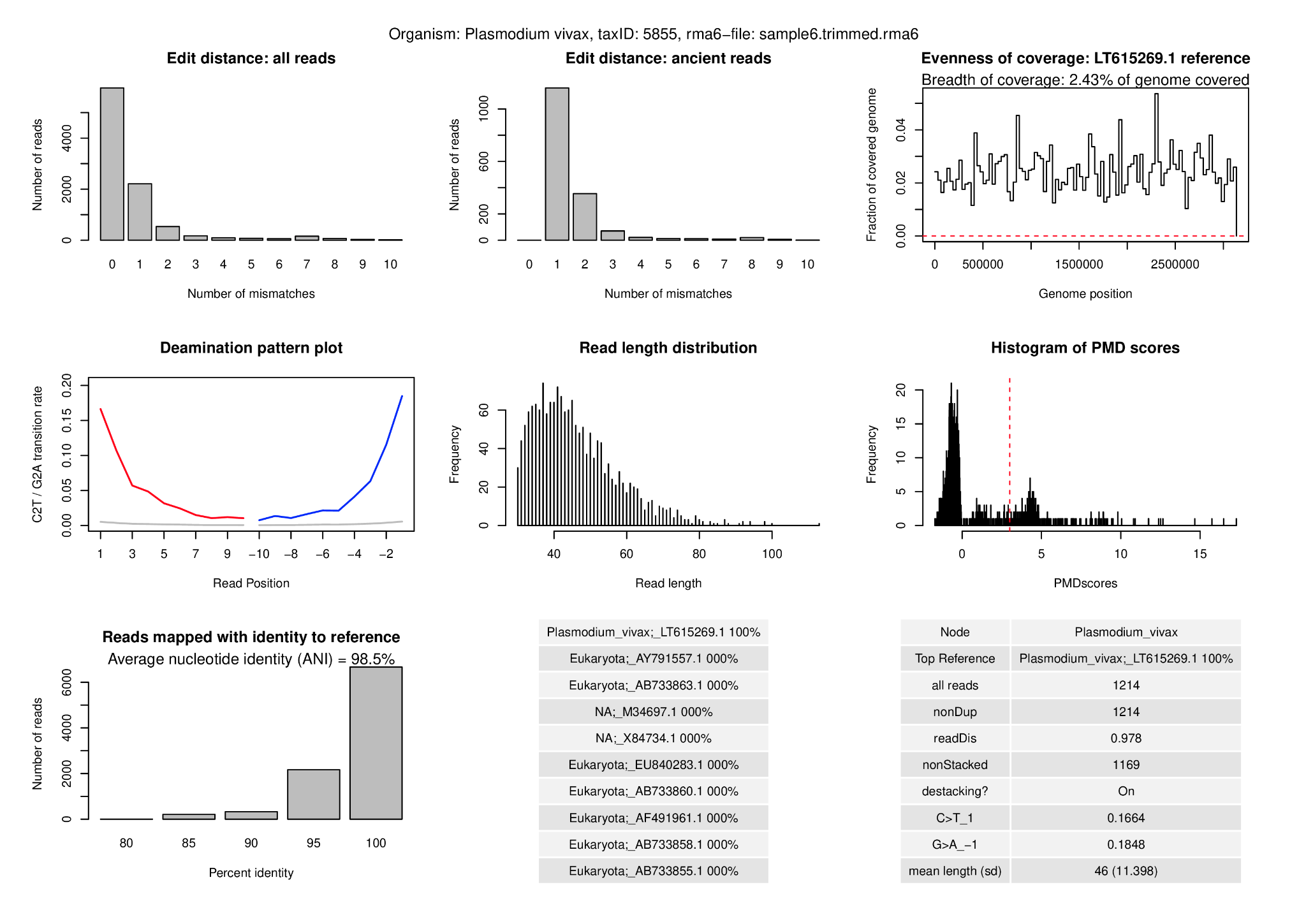


**Fig. S21.** Authentication plot produced by aMeta for *Plasmodium vivax*, an eukaryotic pathogen correctly detected in sample 6 of the simulated pathogen-enriched ancient metagenomic dataset.


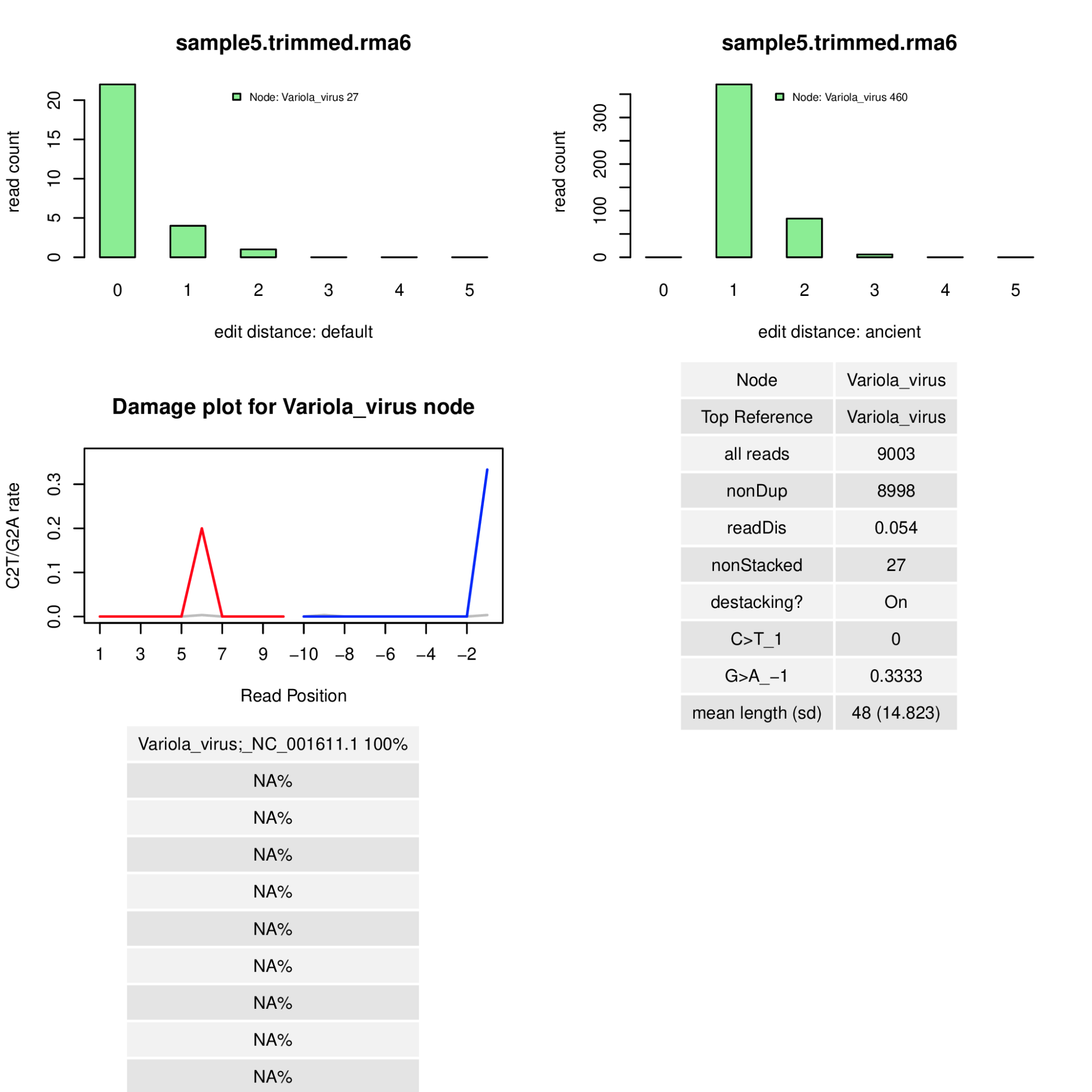


**Fig. S22.** Authentication output from HOPS for *Variola virus* detected for sample 5 from the simulated pathogen-enriched dataset.


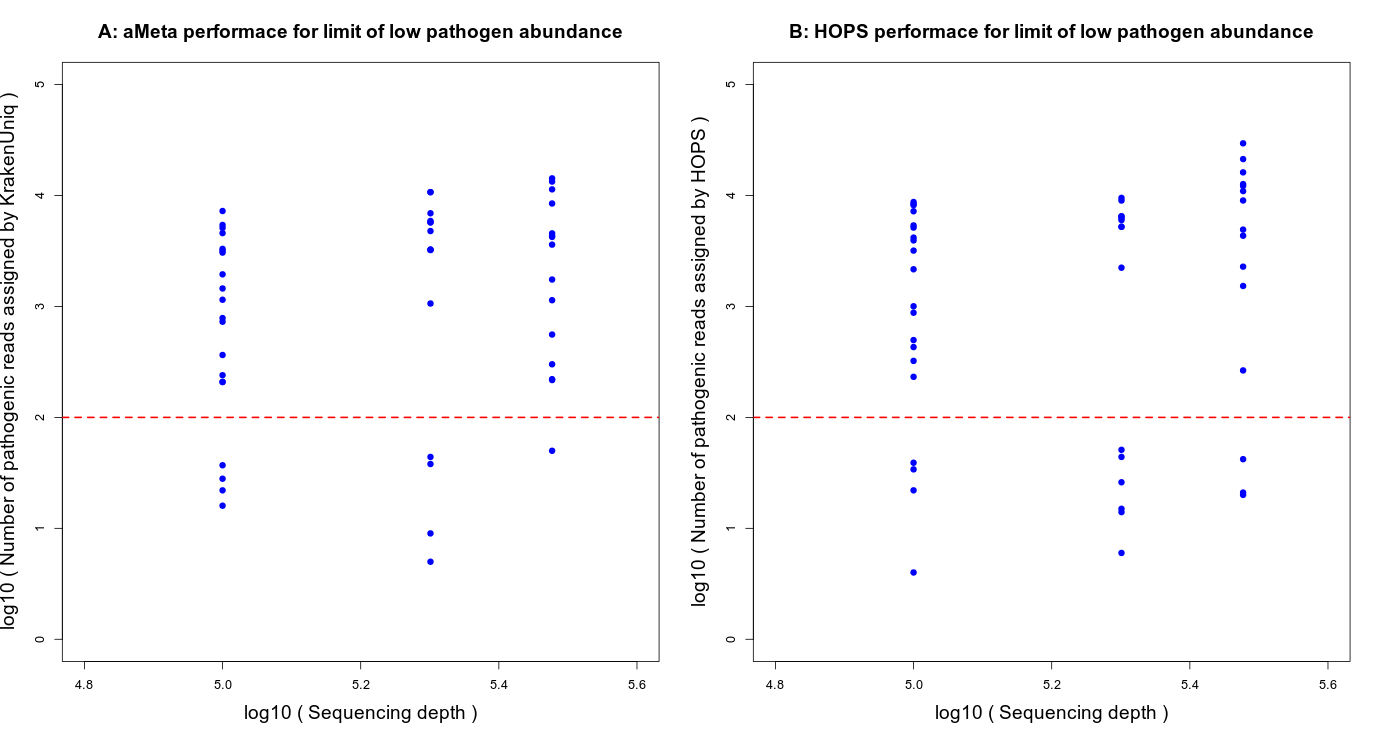


**Fig. S23.** Number of assigned pathogenic reads by A) aMeta, and B) HOPS on simulated low-coverage pathogen-enriched data. The total number of simulated microbial reads varied between 100 000 and 300 000 across the samples. Horizontal dashed lines mark a reasonable read count threshold (~100-300 reads) that can be applied to KrakenUniq and HOPS abundance matrices.


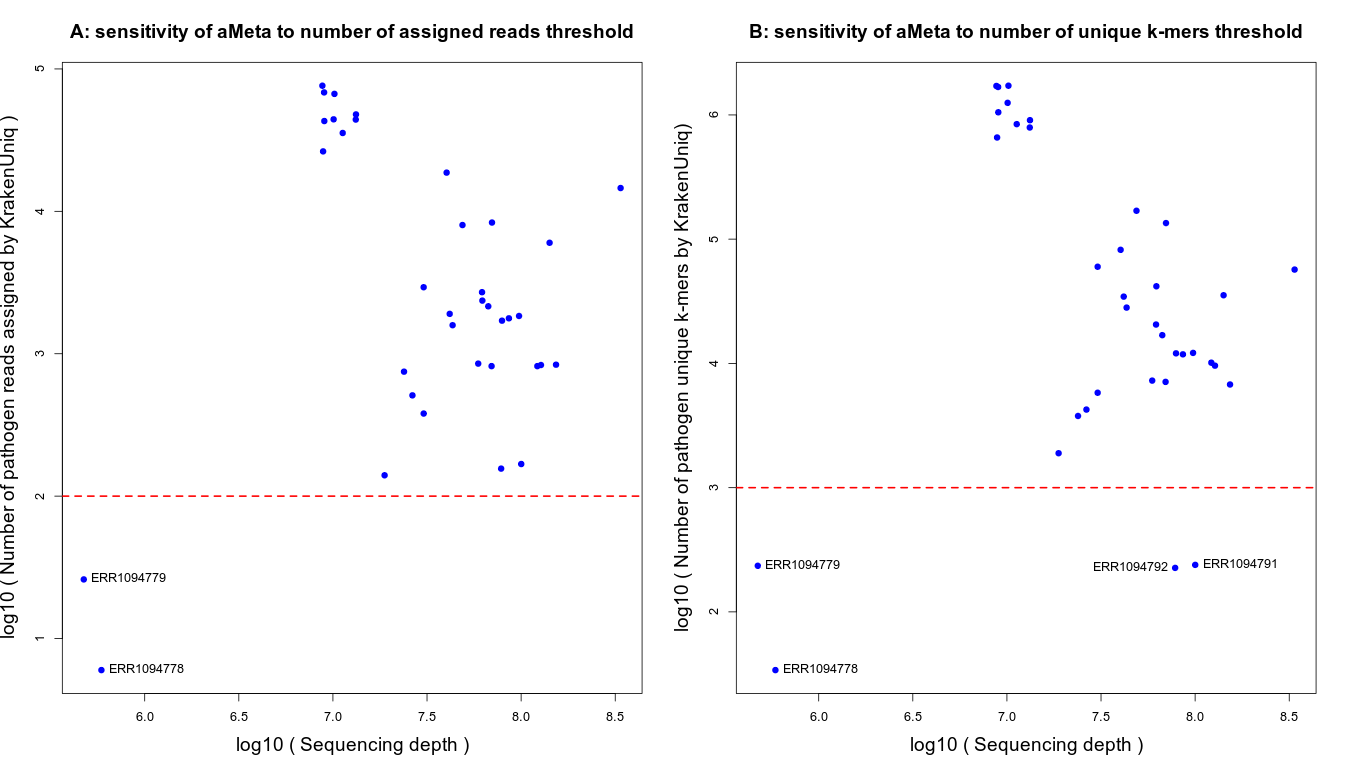


**Fig. S24.** Sensitivity analysis of aMeta with respect to filtering by A) depth (number of assigned reads), and B) breadth of coverage (number of unique *k*-mers) performed on 36 libraries with at least one microbial pathogen reported previously by 4 ancient metagenomic studies [54-57]. The horizontal dashed lines mark default aMeta thresholds: 200 assigned reads and 1000 unique *k*-mers.
