# Supplementary material for: aMeta: an accurate and memory-efficient ancient metagenomic profiling workflow
Source: Genome Biol. 2023 Oct 23;24:242. doi: 10.1186/s13059-023-03083-9 (PMC10591440; doi:10.1186/s13059-023-03083-9)
Supplement: Supplementary file 2 — Additional file 2: Supplementary information S1-S7, other technical details not included in the main text and Methods section [52–56]. [file 13059_2023_3083_MOESM2_ESM.docx]

**aMeta: an accurate and memory-efficient ancient metagenomic profiling workflow**

*Zoé Pochon*^1,2^**, Nora Bergfeldt*^1,3,4^**, Emrah Kırdök*^5^*, Mário Vicente*^1,2,^*, Thijessen Naidoo*^1,2,6^*,
Tom van der Valk*^1,4^*, N. Ezgi Altınışık*^7^*, Maja Krzewińska*^1,2^*, Love Dalen*^1,3^*, Anders Götherström*^1,2^**, Claudio Mirabello*^8^**, Per Unneberg*^9^**, and Nikolay Oskolkov*^10^******^+^***

** shared authorship
+ to whom correspondence should be addressed*^1^ Centre for Palaeogenetics, Stockholm, Sweden

^2^ Department of Archaeology and Classical Studies, Stockholm University, Stockholm, Sweden

^3^ Department of Zoology, Stockholm University, Stockholm, Sweden

^4^ Department of Bioinformatics and Genetics, Swedish Museum of Natural History, Stockholm, Sweden

^5^ Department of Biotechnology, Faculty of Science, Mersin University, Mersin, Turkey

^6^ Ancient DNA Unit, Science for Life Laboratory, Stockholm and Uppsala, Sweden

^7^ Human-G Laboratory, Department of Anthropology, Hacettepe University, 06800, Beytepe, Ankara, Turkey

^8^ Department of Physics, Chemistry and Biology, National Bioinformatics Infrastructure Sweden, Science for Life Laboratory, Linköping University, Linköping, Sweden

^9^ Department of Cell and Molecular Biology, National Bioinformatics Infrastructure Sweden, Science for Life Laboratory, Uppsala University, Uppsala, Sweden

^10^ Department of Biology, National Bioinformatics Infrastructure Sweden, Science for Life Laboratory, Lund University, Lund, Sweden

**Supplementary Information**

**S1. Validation of KrakenUniq results via alignment with Bowtie2**

Taxonomic classification with KrakenUniq delivers a reliable list of organisms present in a sample. However, the detected organisms have to be validated and their ancient status has to be confirmed. Since this is not possible to do with taxonomic classification alone, additional alignment tracking is needed as it can provide information on coverage and damage of detected organisms. Therefore, aMeta implements the alignments of aDNA sequences to their respective reference genomes using Bowtie2 [19] and MALT [20] aligners. The two aligners are different in terms of mapping methods as well as speed and resource demands. While MALT uses the Lowest Common Ancestor (LCA) algorithm that is preferred for metagenomic data, Bowtie2 is a general purpose aligner that does not assign LCA to each aDNA sequence. Nevertheless, we found that an advantage of Bowtie2 is that it was faster and less resource-intensive than MALT. This has enabled the seamless build and use of large Bowtie2 indexes for the full NCBI non-redundant NT database (currently requires 600 GB of RAM), which is publicly available for the scientific community via the SciLifeLab Figshare at <https://doi.org/10.17044/scilifelab.21070063>, as well as for a microbial pathogen-enriched database (“PathoGenome”, requires 256 GB of RAM, it is available at the SciLifeLab Figshare via <https://doi.org/10.17044/scilifelab.21185887>) for fast validation and authentication of potentially present pathogenic species, Figure 1. The latter was built using a custom permissive list of pathogenic microbial organisms derived from the literature. Thus, Bowtie2’s global (end-to-end) alignments can be directly used to compute evenness of coverage, edit distance [23], deamination profile with e.g. mapDamage [27] and other validation and authentication metrics. However, Bowtie2 provides very conservative alignments by not applying LCA. This leads to ignoring aDNA sequences that actually originate from a certain species but cannot be unambiguously attributed to that species due to their length and conservation. These aDNA sequences are marked as multi-mappers by Bowtie2 and are usually removed from downstream analyses. Therefore an additional LCA-based alignment step with MALT [20] is strongly recommended to enhance the robustness of microbiome reconstruction.

**S2. Limitations of MALT and HOPS**

The HOPS pipeline [23] was originally developed for screening ancient metagenomics samples to detect the presence of ancient pathogenic microorganisms via LCA-based alignment against a reference database. However, a truly unbiased microbe / pathogen detection is only possible with a sufficiently large reference database. As discussed in the “Results - Effect of database size” subsection of the main text, if the size of the reference database is not satisfactory, first, microbial organisms truly present in a sample but not included into the reference database may be missed; and second, reads from microbial organisms not included in the reference database may potentially be misaligned to other phylogenetically related species in the database. In other words, the selection of microbial organisms to include in the reference database may severely bias the outcomes of the screening workflow. We found that MALT and HOPS can be prone to this bias due to their inherently limited ability to use large reference databases. Building a database and running HOPS on even a limited number of RefSeq reference genomes, such as complete genomes, can easily require up to 1 TB of computer memory (RAM), imposing severe limitations on users without access to computer clusters with large memory nodes. Ideally, for better diversity of the database, one would like to include at least chromosome or scaffold level assemblies in the database as well, however, in this case, building a MALT database and running HOPS requires computer nodes with RAM far beyond 1 TB, e.g. a full NCBI NT MALT database requires 3.5 TB of RAM, which is not available to most labs. Sometimes, for reducing database size, random sampling per species or genus is implemented, which is obviously not optimal as poor quality reference genomes might be included into the database in this way. In addition, as it is shown in the *Replication on pathogen-enriched simulated and real datasets* sub-section in the main text for the case of *Borrelia recurrentis*, it might be critical for pathogen detection to include all available strains in the database. An alternative way of reducing a MALT database size by prioritizing representatives across a clade (based on average nucleotide identity) does not seem to be straightforward or accurate enough either, as it requires deep domain knowledge and tedious manual picking of good quality references as well as comparing phylogenetic similarities with their neighbors.

Another substantial constraint of HOPS is that it does not provide information on the breadth and evenness of coverage. As discussed in the main text and shown in Figure 2, this often results in erroneous detection of microbial organisms that happen to have a large number of reads aligned to conserved regions of their reference genomes, see Additional file 1: Fig. S2.

Below, we list a few other minor disadvantages of HOPS that can nonetheless severely impact the analysis outcome if the users are not properly informed. First, vanilla HOPS lacks an adapter trimming procedure, so we experienced that a naive usage of HOPS on raw data can lead to erroneous results when very few reads get assigned to reference sequences. However, running HOPS via nf-core / eager takes care of this issue and should be preferred over the original HOPS. Second, both MALT and HOPS work primarily with the *rma6* alignment format which is non-standard in bioinformatics and cannot be easily handled. This leads to a strong need to use MaltExtract, an extension of MALT, to process *rma6* files. However, MaltExtract is not easily customizable and offers a limited set of operations by default. In particular, it accepts scientific names of organisms instead of taxIDs, which often results in failure to handle an organism in question due to altered annotations in the NCBI databases or due to lack of a valid match between the scientific name and taxID. Moreover, MaltExtract can only authenticate a priori known candidates, for example microbial pathogens, i.e. it does not test all detected microbes. Third, HOPS does not provide alignments in a more traditional SAM format although MALT does. However, the SAM-alignments delivered by MALT lack an LCA implementation and in that sense are equivalent to Bowtie2 alignments, nevertheless they cannot be considered as a good replacement for the Bowtie2 alignments since they in turn lack important quality metrics such as MAPQ. In summary, an issue with using MALT / HOPS alignments is that the *rma6* format with LCA is not very bioinformatic-friendly and cannot be processed with standard bioinformatics tools such as SAMtools; on the other hand, SAM-alignments are either not delivered at all (HOPS), or, if delivered, lack LCA and fundamental alignment quality metrics (MALT). Finally, the HOPS scoring system, more specifically, the *postprocessing.AMPS.r* script belonging to the HOPS toolbox, by default assigns +1 to any microbial hit with non-zero terminal damage. Therefore, even a tiny transition mutation frequency, such as 0.001 at the terminal ends of the reads, counts as the presence of damage, which substantially inflates the authentication error, Additional file 1: Fig. S12.

Taking into account the shortcomings of MALT and HOPS, such as large resource demands, limited database size, difficulties with *rma6* output format and limitations with filtering by MAPQ and breadth / evenness of coverage, a new generation of ancient metagenomic specific LCA-based aligners is required. A promising alternative that we suggest for future development is to perform alignments with Bowtie2, which is fast and does not need much computer memory (RAM), and to fine-tune the multi-mapping reads with the recently developed *sam2lca* algorithm [45]. In our opinion, Bowtie2 + sam2lca should entirely replace MALT while delivering alignments in bioinformatics-friendly SAM-format. Other useful metrics provided by HOPS such as deamination pattern and edit distance can easily be computed by mapDamage [27], and by retrieving NM-tag information from the SAM-alignments delivered by Bowtie2.

**S3. Breadth and evenness of coverage computation**

Breadth and evenness of coverage of alignments for each microbial organism in Figure 4 was addressed using the *samtools depth* command from SAMtools with *-a* flag [29]. This command produces a file reporting the number of reads covering each position of the reference genome. The way aMeta calculates the breadth / evenness of coverage plot, for example in Figure 4, is by splitting the reference genome into 100 bins / tiles and computing the number of positions covered by at least one read normalized by the total number of genomic positions within each bin / tile.

It is expected that a good evenness of coverage has few or no bins with a value of zero. Consider the example of *Y. pestis* hit in simulated ancient metagenomic sample 10. In total, there were around 12 000 reads simulated to belong to *Y. pestis* and 11 000 of them mapped uniquely to *Y. pestis* reference genome. Assuming the reads to be roughly 50 bp long on average and almost non-overlapping with each other, they can thus cover at most 11000*50/4600000 ~ 12% of ~4.6 Mbp long *Y. pestis* reference genome. The evenness of coverage is then computed within 100 bins. Since, in this particular *Y. pestis* within sample 10 example, the reads are aligned uniformly across the reference, there are at least a few reads within each bin. Therefore, there are no totally empty bins, otherwise, that would result in dropping the evenness of coverage profile strictly to 0. Note, that within each bin the breadth of coverage is not high, it is around 12%, i.e. only ~120 bp out of 1000 bp are covered at least once. However, the breadth of coverage is nowhere 0, which would mean uneven coverage. Note, that due to the nature of the SAM-output from the MALT tool, secondary alignments or multi-mapping reads can contribute to the breadth / evenness of coverage plot, which might potentially inflate the true coverage but is difficult to address within the current MALT implementation. On the other hand, omitting the multi-mapping ambiguous reads from breadth / evenness of coverage computation is not optimal either, as the reads might very well truly originate from the organism of interest, and therefore these reads should be taken into account when computing breadth / evenness of coverage unless a very conservative analysis is the goal. Empirically, testing aMeta in different projects we found that the breadth / evenness of coverage plot, despite being permissive, provides very valuable additional validation and authentication information.

**S4. Computing detection errors of aMeta and HOPS**

Additional file 1: Fig. S5 demonstrates a ground truth microbial abundance heatmap across the 10 simulated samples. The elements of the matrix correspond to the simulated fraction of a microbe in a metagenomic sample. The ground truth abundance matrix was binarized using 1% of all reads as the detection threshold, and a binary (0 - absent / 1 - present) heatmap of the distribution of microbial organisms in the samples is presented in Additional file 1: Fig. S6. To compare the microbial detection accuracy of HOPS and aMeta, we built binary heatmaps based on the microbial abundances reconstructed by aMeta and HOPS, see Additional file 1: Fig. S7 and S8, respectively, and compared them with the ground truth in Additional file 1: Fig. S6. For aMeta, the default internal KrakenUniq filter settings (1000 unique *k*-mers and 200 assigned reads) were used to determine the presence / absence of a microbe, while we used a threshold of 200 reads to binarize the HOPS microbial abundance matrix and match it with aMeta. Nevertheless the exact threshold value does not significantly affect the conclusions as we show later. From Additional file 1: Fig. S6, S7 and S8, one can conclude that both HOPS and aMeta failed to reconstruct the following 7 simulated species: *Acanthamoeba castellanii, Aspergillus flavus, Bradyrhizobium erythrophlei, Mycolicibacterium aurum, Planobispora rosea, Prosthecobacter vanneervenii,* and *Vermamoeba vermiformis.* These species were either absent from the reference databases or their abundance was below the detection thresholds in all samples. However, it is clear that the sensitivity of HOPS was poorer than that of aMeta, as HOPS additionally missed the following 9 species: *Campylobacter rectus*, *Fusarium fujikuroi*, *Methylobacterium bullatum*, *Micromonas commoda, Micromonospora echinospora*, *Mycobacterium riyadhense*, *Nonomuraea gerenzanensis*, *Pseudomonas psychrophila*, and *Pseudomonas thivervalensis* in all simulated samples while aMeta detected the species correctly in some of the samples. In total, HOPS missed 16 out of 35 microbial species, while aMeta missed only 9 out of 35. Therefore, completely undetected species contribute to the very high false negative detection rate of HOPS. In addition, the specificity of HOPS was lower than that of aMeta. For example, microbial species such as *Mycobacterium avium, Nocardia brasiliensis, Rhodopseudomonas palustris, Sorangium cellulosum* and *Streptosporangium roseum* were incorrectly detected by HOPS in at least one simulated metagenomic sample, while they did not pass aMeta filtering and were correctly excluded for these samples, see Additional file 1: Fig. S6, S7 and S8.

To summarize the results, we computed the accuracy of microbial detection, i.e. presence or absence irrespective of ancient status, based on the confusion matrices for aMeta and HOPS, see Additional file 1: Fig. S9. The confusion matrix corresponding to aMeta had a detection accuracy of 80% and was more balanced than the HOPS confusion matrix, which had an accuracy of 69% and a very high false negative rate. While a comparable number of false positive discoveries were reported by HOPS (12) and aMeta (9), the false negative rate of HOPS was ~1.5 times higher (96) than that of aMeta (60), resulting in an overall accuracy of microbial detection by aMeta that was much higher than that of HOPS. It is important to point out that the higher accuracy of microbial detection from aMeta is not due to inappropriate filters, i.e. the 200 reads threshold applied to the HOPS microbial abundance matrix, but to the fact that many species were not present in the HOPS database (built based on the NCBI RefSeq database of complete microbial genomes). Note that despite this missingness, the NCBI RefSeq database of complete microbial genomes used by HOPS required almost twice the memory resources (RAM) compared to aMeta, see Figure 5. To assess the effect of HOPS filtering, we checked the range of different numbers of assigned reads (depth of coverage), with thresholds ranging from 0 (no filtering) to 800 reads (very harsh filtering), see Additional file 1: Fig. S10. Over the range of read count thresholds tested, the microbial detection accuracy of HOPS was consistently lower than that of aMeta (Additional file 1: Fig. S10A), while the amount of false positive and false negative counts was consistently higher for HOPS than for aMeta (Additional file 1: Fig. S10B and S10C).

We would like to explicitly emphasize again that the difference in detection sensitivity (not in specificity, authentication and resource usage) between aMeta and HOPS comes to a large extent from, strictly speaking, non-identical databases used for the comparison. This, in turn, comes from the fact that it was technically impossible to build and utilize a full microbial NCBI NT database with MALT using 1TB of RAM on a computer cluster, however aMeta used less than 400 GB for dynamically building and using the same full microbial NCBI NT database on the same cluster node. Therefore, utilizing identical databases for a perfect comparison of detection sensitivity between aMeta and HOPS would imply using very small databases, which we on purpose tried to avoid in this study, taking into account the high false discovery rate associated with small databases, Figures 8 and 9. We hypothesize that providing large memory resources (over 1 TB of RAM) and using perfectly identical large and diverse databases would likely show a smaller difference in sensitivity between aMeta and HOPS, however this has not been tested in this study.

We also aimed to address aMeta performance in comparison with HOPS on read level. Additional file 1: Fig. S11 compares the number of reads reconstructed by aMeta and HOPS to the ground truth, and specifically explores the performance of aMeta in function of the number of microbial reads detected. Unlike HOPS, which has a high dropout rate (high number of false positives and false negatives) and is therefore sensitive to the read number threshold (meaning that lowering the ~100-300 reads threshold results in higher number of false positives), aMeta is more robust over a wide range of read number thresholds due to the additional breadth of coverage filter that reduces the dropout effect. In other words, lowering the ~100-300 reads detection threshold slightly improves the agreement of aMeta with the ground truth without bringing too many false positive hits. However, reducing the threshold substantially complicates subsequent authentication.

**S5. Scoring systems of aMeta and HOPS**

The scoring system of aMeta represents a sum of eight validation and authentication metrics computed on MALT LCA-based alignments: 1) deamination profile, 2) evenness of coverage, 3) edit distance for all reads, 4) edit distance for damaged reads, 5) read length distribution, 6) PMD scores distribution, and 7) number of assigned reads (depth of coverage), 8) average nucleotide identity (ANI). Each metric can add +1 to the total sum except for the evenness of coverage that can add +2, as it is the most crucial to validate microbial presence, and deamination profile, which can add up to +2 (both 5’ and 3’ ends vote independently), as it is the ultimate criterion for ancient status. Therefore, the range of scores a microbe can obtain varies from a minimum of 0 to a maximum value of 10. Below we explain how each validation and authentication metric presented in Figure 4 is quantified according to the aMeta scoring system.

First, the deamination profile represents an increased frequency of transition mutations (C→T and G→A) at the terminal ends of the sequencing reads compared to the frequencies of all other possible mutations. The scoring system of aMeta adds +1 to the total score if the frequency of C→T exceeds 0.05 at 5’ end and +1 if the frequency of G→A exceeds 0.05 at 3’ end. The exact thresholds can be adjusted depending on the age and preservation conditions of the samples. However we have found that the default thresholds provide sufficient accuracy in most typical cases [35, 36]. Second, the evenness of coverage metric can add +2 to the total sum in the case where the aDNA reads cover the reference genome uniformly. The large contribution of evenness of coverage to the total score is due to its importance for validating the presence of a microbe in a sample. To quantify the evenness of coverage in Figure 4, we split the reference genome into 100 tiles and count the number of tiles with near zero average breadth of coverage (percent of covered bases in a tile). If fewer than 3 tiles have an average breadth of coverage of less than 1%, i.e. if a few aDNA reads are present in almost all tiles, the coverage is considered sufficiently uniform and the total score is increased by 3 points. Note that the mean breadth of coverage across the whole genome may be quite low in this case, for example, only 2-4%. This may reflect the overall low sequencing depth in the case a sample underwent a shallow aDNA sequencing. However, what is most important is that there are reads (even very few) present in nearly all regions of the reference genome. This is what the evenness of coverage metric aims to address. Third and fourth, the edit distance for all reads and damaged reads can add +1 point each, provided that they both have a decreasing profile of the numbers of mapped reads as the number of mismatches increases. This metric controls that the majority of aDNA reads map with no or very few mismatches (damaged reads must have at least one mismatch) by analogy with HOPS [23], and therefore ensures that the reads are mapped to a correct reference genome. We examine the monotonous decline in the numbers of mapped reads by checking that it is always greater for smaller mismatch values. Fifth, the fragmentation of aDNA is inspected via the read length distribution. If 90% of the reads are less than 100bp in length, which ensures that the mode of the read length distribution is located at smaller values typical for aDNA, this quality metric adds +1 to the total score. Sixth, an alternative to the deamination profile assessment of post-mortem damage (PMD) can be performed via PMDtools [31] which computes a likelihood of being ancient for each DNA read. As stated in the original paper [31], a PMD score greater than 3 implies a high likelihood that a read is of ancient origin. This quality metric adds +1 to the total authentication score if at least 10% of the reads have a PMD score above 3. Seventh metric, the depth of coverage is controlled by a 200 DNA reads threshold, which is an empirical number of reads sufficient to compute a statistically reliable deamination profile. If there are more than 200 reads mapped to a reference genome, this adds +1 to the total score. Eights and last, the average nucleotide identity (ANI) estimates the similarity of the mapped reads with respect to the reference sequence. It is derived from the percent identity barplot in Figure 4, and computed as weighted average of identity bins with the numbers of reads in each bin as weights. The average nucleotide identity (ANI) metric can add +1 to the authentication score if it is greater than 95% identity as suggested in [46].

The native HOPS scoring system includes only three quality metrics: deamination profile and edit distances for all reads and damaged reads. This not only results in many obvious false positive discoveries, as shown in Additional file 1: Fig. S12, but also too little variation in scores that is not straightforward to align with aMeta’s eight-metric scoring system to compute a ROC-curve. Therefore, for a proper comparison, we had to modify the native HOPS scoring system and add reasonable assessments of depth and evenness of coverage, as well as PMD scores and read length distribution. The deamination profile metric can still, by analogy with aMeta, add a maximum of +2 to the total HOPS score if the transition frequencies at the terminal ends of the aDNA reads are greater than 0, i.e. if any deamination (not necessarily a strong one) is present. Note that this threshold of 0 is designed by HOPS and hard-coded within the *postprocessing.AMPS.r* script belonging to the HOPS pipeline. The edit distances of all reads and damaged reads can also contribute +2 as long as they have declining profiles. Those three native HOPS metrics are not modified by us at all, but only scored accordingly to agree with the scoring system of aMeta. Next, HOPS actually provides read length distribution, average nucleotide identity (ANI) and depth of coverage metrics but does not use them for its native scoring system. For a more correct comparison with aMeta, we added the read length distribution, average nucleotide identity (ANI) and depth of coverage metrics to the total HOPS score and quantified them in exactly the same way as within the aMeta scoring system. Further, since the evenness of coverage filter is completely absent from HOPS (*readDis* reported by HOPS is not used for filtering, and can not be considered as an optimal proxy for breadth of coverage), it could have been naturally quantified such that HOPS assigns a score of +2 to any microbe, regardless of whether it has uniform coverage or not. However, extending the HOPS scoring system in this manner would obviously worsen its performance. Therefore, based on the strong correlation between depth and breadth of coverage generally assumed until recently in the ancient metagenomics community for the reason explained in Figure 3, we assigned +2 score if there were more than 200 reads aligned to a microbe by HOPS in order to resemble aMeta’s scoring system. Finally, since HOPS does not compute PMD scores, we tried to reasonably add it to the HOPS score by postulating that this quality metric can contribute +1 to the total score if the transition frequency at both terminal ends of the reads exceed 0.05. In summary, the native HOPS scoring system included 3 quality metrics (deamination profile, edit distance of all reads, edit distance of damaged reads) and we left them unchanged. Three other quality metrics (read length distribution, average nucleotide identity and depth of coverage) were straightforward to add since HOPS actually computes them even if it does not use them for scoring, so we added these three quality metrics in exactly the same way as within aMeta. The only two quality metrics that were not provided by HOPS at all, evenness of coverage and PMD score distribution, were added to the total HOPS score in the most reasonable and fair way we could think of. Note that generalizing the native HOPS scoring in order to match the aMeta scoring does not worsen but rather improves its performance over the original HOPS authentication scoring, which is more prone to a high false positive rate.

We used the aMeta scoring system to predict the ancient status of each microbe in the 10 simulated samples, resulting in 174 predictions as scores varying from 0 to 10. By analogy, we used the modified HOPS scoring for each microbe detected by HOPS and obtained 97 predictions. The different number of predictions between aMeta and HOPS can be explained by a better sensitivity of aMeta described in the main text and Additional file 2: S4. The predictions obtained by aMeta and HOPS were compared to the simulated ground truth ancient / modern labels which allowed computing sensitivity versus specificity ROC-curves of microbial authentication.

Finally, aMeta is capable of generating a graphical overview (heatmap) of the authentications scores for each sample and each detected microbe, Additional file 1: Fig. S4. The heatmap demonstrates microbial species (in rows) authenticated for each sample (in columns). The colors and the numbers in the heatmap represent authentications scores, i.e. numeric quantification of seven quality metrics that provide information about microbial presence and ancient status. The authentication scores can vary from 0 to 10, the higher is the score the more likely that a microbe is present in a sample and is ancient. Typically, scores from 8 to 10 (red color in the heatmap) provide good confidence of ancient microbial presence in a sample. Scores from 5 to 7 (yellow and orange colors in the heatmap) can imply that either: a) a microbe is present but not ancient, i.e. modern contaminant, or b) a microbe is ancient (the reads are damaged) but was perhaps aligned to a wrong reference, i.e. it is not the microbe you think about. The former is a more common case scenario. The latter often happens when an ancient microbe is correctly detected on a genus level but we are not confident about the exact species, and might be aligning the damaged reads to a non-optimal reference which leads to a lot of mismatches or poor evenness of coverage. Scores from 0 to 4 (blue color in the heatmap) typically mean that we have very little statistical evidence (very few reads) to claim presence of a microbe in a sample.

**S6. Snakemake implementation of aMeta**

We implemented the pipeline with the Snakemake workflow management system [24]. The workflow together with installation instructions, documentation and test data set is available at [https://github.com/NBISweden/aMeta](https://github.com/NBISweden/ancient-microbiome-smk) [58] and <https://zenodo.org/record/8354933> [59]. The following section gives a brief overview of the implementation, configuration and execution details of the Snakemake workflow.

We followed the best practice guidelines [47] that structure the workflow repository according to the Snakemake workflow template [48] that organizes workflow-related files in a *workflow* directory, and configuration files in a *config* directory. Briefly, workflow command lines, so-called rules, are placed in a *workflow / rules* directory arranged by topics. The files consist of Snakemake codes that define operations on inputs to generate outputs, where the workflow manager determines dependencies between the rules. The modular rules files are connected via a *workflow / Snakefile*, which serves as the main entry point for the workflow. Custom scripts that are executed via rules are included in *workflow / scripts*. In order to enable reproducibility, each rule defines isolated software environments that are deployed with the *conda* package manager [49]. *Conda* environment files are stored in *workflow / envs*. The workflow configuration also supports the use of environment modules commonly used in high performance cluster systems HPC.

In addition to the workflow execution files described above, the directory *workflow / schemas* stores configuration *schemas* that define sample data and configuration file formats. *Schema* validation is applied to ensure the validity of sample sheets and configuration files. The configuration file mainly defines sample-sheet location and database resources. An example of a configuration file and a sample sheet can be found in the GitHub repository of the workflow at [https://github.com/NBISweden/aMeta](https://github.com/NBISweden/ancient-microbiome-smk) [58] and <https://zenodo.org/record/8354933> [59].

The general advice for running the workflow is to clone the repository and organize data files following the directory structure guidelines. Then, given a configuration file *config / config.yaml* and sample sheet, the workflow can be executed from the root directory of the repository. Compute resource usage, such as memory and run time, can be further fine-tuned on a rule-by-rule basis through the use of *snakemake profiles* [50].

As mentioned above, the execution order of different Snakemake rules is determined by the workflow manager creating a Directed Acyclic Graph (DAG) of jobs that can be automatically parallelized. A typical DAG of aMeta run is presented in Additional file 1: Fig. S17.

**S7. Other technical details**

Below we provide a few unrelated but presumably important technical details about our analysis.

*Details on computing Figure 8*

When testing for the effect of database size and spurious misalignments that can potentially arise for small databases, we used a random metagenomic stool sample from a modern infant who unlikely suffered from plague, and therefore no *Y. pestis* was expected to be found in this sample. The metagenomic fastq-file with ID G69146_pe_1.fastq.gz was downloaded from the DIABIMMUNE metagenomic database, Three Country Cohort [37], <https://diabimmune.broadinstitute.org/diabimmune/three-country-cohort/resources/metagenomic-sequence-data>, and aligned to *Yersinia pestis (Y. pestis)* CO92 reference genome alone, <https://www.ncbi.nlm.nih.gov/genome/153?genome_assembly_id=299265>. Next, we concatenated the *Y. pestis* CO92 reference genome with the hg38 version of human reference genome, <https://hgdownload.soe.ucsc.edu/goldenPath/hg38/bigZips/>.

*Details on running comparison with HOPS*

Sequencing adapters were removed prior to the HOPS run, as this step is not implemented in HOPS by default. To quantify microbial organisms from the output *rma6*-files of HOPS, the *rma2info* command from MEGAN tool [32] and its wrapper, rma-tabuliser, developed by James A. Fellows Yates [51] were used.

*Details on default aMeta filters*

By default we filter output of KrakenUniq using 1000 unique *k*-mers (breadth of coverage filter), and 200 reads (depth of coverage filter) assigned to microbial species. Therefore, the dashed horizontal line in Figure 6 corresponds to the IoU and F1 score computed using the default depth (200 assigned reads) and breadth (1000 unique *k*-mers) of coverage thresholds set in aMeta. Nevertheless, the users are encouraged to experiment with assigned reads threshold in the range of ~100-300 reads, and unique *k*-mers threshold in the range of ~500-1500 *k*-mers.

*Details on computing Figure 6*

F1 score was computed according to the formula F1 = (2 * TP) / (2 * TP + FP + FN), while the IoU metric, aka Jaccard similarity, was computed as IoU = length of intersection between prediction and ground truth / length of union between prediction and ground truth.

*Details on computing Figure 7*

ROC-curve was computed from aMeta and HOPS scores by using *rocit* function from *ROCit* R package https://cran.r-project.org/package=ROCit with *binormal* method.

*Details on multi-threading used in aMeta*

An additional speed advantage of aMeta comes from optimization of all steps with GNU parallel [52] that is extensively used, for example, for computing deamination profiles with mapDamage [27] in parallel for a number of microbial organisms.

*Details on the replication analysis with pathogen-enriched simulated dataset*

The additional 10 samples enriched for pathogens were simulated with the gargammel tool [34] using the same command lines as described in the Additional file 2: S4. However, this time, we specifically addressed the limit of very low pathogen abundance by significantly restricting the number of microbial reads to occupy at most 30% of all reads, while previously, in the main analysis, this fraction varied in a wide range from 30% to 70% (the remainder being human reads). More specifically, the following pathogen fractions for the samples 1 to10 were used for simulating the pathogen-enriched dataset: 0.1, 0.1, 0.1, 0.1, 0.2, 0.2, 0.2, 0.3, 0.3, 0.3. In total, there were 9 ancient pathogens and 4 modern bacterial contaminants simulated:

*Ancient:* *Brucella melitensis, Hepatitis B virus, Human parvovirus B19, Mycobacterium leprae, Mycobacterium tuberculosis, Plasmodium vivax, Treponema pallidum, Variola virus, Vibrio cholerae.*

*Modern: Burkholderia mallei, Pseudomonas caeni, Pseudomonas psychrophila, Pseudomonas thivervalensis.*As in the main analysis, in total 500 000 ancient and 500 000 modern reads were simulated per sample. Next, aMeta and HOPS with default settings were executed on the pathogen-enriched dataset. The ground truth, detection and authentication outcomes from aMeta and HOPS are presented in Additional file 1: Fig. S18, S19 and S20, respectively. The simulated dataset enriched for microbial pathogenic species is available at <https://doi.org/10.17044/scilifelab.24211584>.
